# Diagnostic Workup, Treatment Patterns, and Clinical Outcomes in Early-Stage IB–IIIA Non-Small-Cell Lung Cancer Patients in Denmark

**DOI:** 10.3390/cancers15215130

**Published:** 2023-10-25

**Authors:** Ebbe Meldgaard Uldbjerg, Lars Ringgaard, Klaus Kaae Andersen, Line Elmerdahl Frederiksen, Aleksandar Jovanovic, Peter Meldgaard

**Affiliations:** 1 Department of Oncology, Aarhus University Hospital, 8200 Aarhus, Denmark; 2 AstraZeneca, 1799 Copenhagen, Denmarkline.frederiksen@astrazeneca.com (L.E.F.); 3 Omicron ApS, 2200 Copenhagen, Denmark; 4 Department of Experimental Oncology, Aarhus University Hospital, 8200 Aarhus, Denmark; 5 Institute for Clinical Medicine, Aarhus University Hospital, Palle Juul-Jensens Boulevard 35, 8200 Aarhus, Denmark

**Keywords:** non-small-cell lung cancer, real-word evidence, treatment, epidermal growth factor receptor mutation status, survival, relapse

## Abstract

**Simple Summary:**

This real-world evidence study contributes important insights for clinicians to further optimize treatment options and improve clinical outcomes in patients with early-stage non-small-cell lung cancer (NSCLC), considering evolving clinical trials on new perioperative treatment modalities. This Danish register-based study followed 1341 stage IB-IIIA patients diagnosed with NSCLC in Aarhus University Hospital between 2010 and 2021. Half of all patients received surgery and 9% stereotactic body radiation therapy. EGFR testing was performed in 42% of patients, of whom 10% had an EGFR mutation. Filling an important evidence gap, this study reported a substantial increase in disease-free survival (DFS) from 2010 to 2020 and across different treatment modalities. Five-year DFS was 49%, 42%, and 22% for stage IB, II, and IIIA patients, respectively. Despite these improvements, disease relapse remained a challenge, with approximately 40% of stage IIIA having relapsed 3 years since diagnosis, highlighting the need for improved treatment strategies.

**Abstract:**

Despite recent improvements in early-stage non-small-cell lung cancer (NSCLC), disease relapse remains challenging. Moreover, real-world evidence on long-term follow-up of disease-free survival (DFS) and recurrence patterns in a large, unselected cohort of early-stage NSCLC patients is lacking. This cohort study aimed to assess clinical characteristics, diagnostic workup, treatment, survival, and risk of disease relapse among early-stage NSCLC patients. Adult patients with stage IB, II, or IIIA NSCLC diagnosed and/or treated at Aarhus University Hospital in Denmark from January 2010 to December 2020 were included and followed-up until May 2021. Comprehensive clinical data were collected from electronic medical records of eligible patients and linked to Danish register data. The study population comprised 1341 early-stage NSCLC patients: 22%, 40%, and 38% were diagnosed with stage IB, II, and IIIA disease, respectively. In total, 42% of patients were tested for epidermal growth factor receptor (EGFR), of whom 10% were EGFR-mutation-positive (EGFRm^+^). Half of all patients received surgery, and nine percent of patients received stereotactic body radiation therapy (SBRT). Disease-free survival 5 years post-diagnosis was 49%, 42%, and 22% for stage IB, II, and stage IIIA patients, respectively. DFS improved over time both for patients treated with surgery and SBRT. However, disease relapse remained a challenge, with approximately 40% of stage IIIA having relapsed 3 years post-diagnosis. This study contributes important knowledge that puts clinical trials on new perioperative treatment modalities for early-stage NSCLC patients into perspective. Our findings cover an essential evidence gap on real-world DFS and recurrence dynamics, confirming that despite an improvement in DFS over time and across different treatment modalities, disease relapse remains a monumental challenge. Therefore, better treatment strategies are needed.

## 1. Introduction

In Denmark, about 5000 patients are diagnosed with lung cancer each year [1,2]. Non-small-cell lung cancer (NSCLC) is the most frequently diagnosed subtype of lung cancer, representing roughly 85% of all lung cancer cases [3,4,5] and with an incidence that continues to increase worldwide [6].

Although survival has considerably improved over the past decades, 5-year survival rates remain low [2]. Increased treatment options and early detection of NSCLC remain the best way to improve survival probability; however, only 35% of patients with NSCLC are diagnosed at an early stage [4]. Recent data from 20 countries suggest that the smallest, localized tumor lesions in stages IA1 and IA3 have 5-year overall survival (OS) probabilities of 92% and 77%, respectively [7,8]. Increasing tumor size and lymph node involvement further decreases survival chances, and the 5-year survival is reduced to less than 10% in the presence of a distant metastasis [7,8]. In the early-stage NSCLC setting, it has been shown that DFS correlates with OS both in clinical trials and observational cohorts [9]. Importantly, DFS data on long-term follow-up and dynamics of recurrence in a real-world evidence setting are not well characterized in a large unselected cohort of stage IB-IIIA NSCLC patients.

Surgical treatment with curative intent is the cornerstone for treating early-stage NSCLC patients in combination with postoperative adjuvant treatment options [10,11,12]. However, treatment options are highly dependent on patient characteristics, stage of disease, tumor location, and tumor-associated biomarkers [6]. Other curative-intended treatment options for patients with early-stage NSCLC include stereotactic body radiation therapy (SBRT) for stages I and II [13] and chemoradiation in combination with immunotherapy for stage III NSCLC [12,14,15]. 

Additionally, driver mutations play an important role in the oncogenesis of NSCLC, including epidermal growth factor receptor-positive mutations (EGFRm^+^). The ADAURA study has demonstrated that adjuvant treatment with osimertinib (a third-generation EGFR tyrosine kinase inhibitor) following complete tumor resection in patients with EGFRm^+^ stage IB-IIIA NSCLC results in a statistically significant and clinically meaningful prolongation of disease-free survival (DFS) compared to the placebo [16,17]. Based on these data, international treatment guidelines now recommend adjuvant osimertinib in this patient population [15,18].

With the emergence of new treatment options for early-stage NSCLC patients, the aim is to diminish the monumental challenge of the high risk of disease relapse [10,11]. Moreover, novel treatments highlight the unmet need associated with optimizing patient selection in the perioperative setting. Long-term follow-up of early-stage NSCLC patients and identification of what characterizes patients who experience disease relapse need further investigation.

This study aims, therefore, to describe the clinical characteristics and diagnostic workup among early-stage (IB-IIIA) NSCLC patients, their initial treatment pattern, overall survival, DFS, and risk of disease relapse in a Danish cohort of patients diagnosed with early-stage NSCLC.

## 2. Materials and Methods 

### 2.1. Study Design and Data Sources

This was a retrospective, observational cohort study of early-stage NSCLC patients diagnosed and/or referred for treatment at a single-site, a university hospital in Denmark. Data were extracted from electronic medical record reviews of eligible patients by participating physicians (or the physician’s delegated clinical research staff) using an electronic data collection form to obtain information on patient characteristics, diagnostic workup, treatment modalities, and clinical outcomes. All individual-level data were subsequently linked to nationwide register data on vital status and emigration status from the Danish Civil Registration System. A unique personal identification number assigned to all individuals resident in Denmark enabled accurate individual-level linkage across these data sources.

### 2.2. Study Population and Outcomes

Individuals aged ≥18 years and diagnosed with stage IB, II, or IIIA NSCLC who had been diagnosed at and/or referred to the Department of Oncology at Aarhus University Hospital for cancer treatment between 7 January 2010 and 11 December 2020 were included in this study.

Information on patient characteristics, diagnostic workup, treatment modalities, and clinical outcomes was captured for all eligible patients diagnosed with stage IB-IIIA NSCLC. The diagnostic workup included the date of diagnosis, EGFR-sensitizing mutation status (EGFRm^+^/no EGFR mutation), programmed death-ligand 1 (PD-L1) expression level (0–1, 1–50, >50) based on the 22C3 immunohistochemical assay, histology (adenocarcinoma, other), performance status (0, 1, 2+), and disease stage (IB, II, IIIA) classified by tumor size and spread to local lymph nodes according to the 7th edition of the AJCC system and subsequently updated to the 8th edition.

Initial treatment modalities were categorized into surgery (no/yes), chemotherapy (no/yes), and SBRT (no/yes). Stereotactic body radiation therapy was administered to patients who were either inoperable or had progressed with oligometastases following surgery. Chemotherapy was only given as adjuvant treatment since neoadjuvant chemotherapy was not the standard of care for early-stage NSCLC patients in the Nordic region during this study. Patients were regarded as having received palliative treatment if they did not receive any oncology-related treatment modalities (i.e., surgery, chemotherapy, or SBRT) at any time since their NSCLC diagnosis. Additionally, the occurrence of metastasis in the central nervous system (CNS) (no/yes), as well as disease relapse and location of spread (local, regional, distant), were captured during follow-up.

### 2.3. Statistical Analyses

Patient follow-up started on the date of NSCLC diagnosis (i.e., index date) and ended on the date of emigration, death, loss to follow-up, or study end on 27 May 2021, whichever occurred first.

Patient and tumor characteristics were described using median and interquartile range (IQR) for continuous variables and counts and proportions for categorical variables. Descriptive statistics were presented stratified by disease stage at diagnosis, i.e., stages IB, II, and IIIA. Differences in baseline characteristics were compared across disease stages with *p*-values reported based on the Chi-square test for categorical variables and ANOVA test for continuous variables. For continuous variables not assumed to be normally distributed, we used the Kruskal–Wallis non-parametric test. Absolute numbers and proportions of patients receiving different treatment modalities were visualized using UpSet plots.

Clinical outcomes included assessment of OS, DFS, disease relapse, and death during follow-up. We defined OS as the time from date of diagnosis until date of death from any cause, whereas DFS was defined as time from date of diagnosis until the date of disease relapse or date of death from any cause. Using time-to-event methods, the risk of death, disease relapse, or being disease-free by time since diagnosis was estimated using the crude cumulative incidence function accounting for competing risks and presented by disease stage (IB, II, IIIA) and treatment modality (surgery with or without chemotherapy vs. SBRT with or without chemotherapy). Similarly, the risk of disease relapse and location of relapse (local, regional, distant) was estimated using the cumulative incidence function and presented by disease stage (IB, II, IIIA) and treatment modality (surgery with or without chemotherapy vs. SBRT with or without chemotherapy).

Disease-free survival was estimated by disease stage (IB, II, IIIA) and treatment modality (surgery with or without chemotherapy vs. SBRT with or without chemotherapy) and presented as Kaplan–Meier plots. To assess improvements in disease-free survival during the study period, DFS estimates were also stratified by year of diagnosis (2010–2013, 2014–2017, 2018–2020) and presented as Kaplan–Meier plots. To adjust for possible confounding, DFS was further evaluated using a Cox proportional hazards model. The time scale used was time since date of diagnosis. Analyses were based on complete cases, i.e., observations with missing values were excluded. Case-wise deletion was considered appropriate since only a small number of patients (<5%) had missing values in one or several variables. All estimates from the Cox proportional hazards model were presented in a forest plot with 95% confidence intervals (CIs).

The statistical software R v. 4.2.1 [19] was used for all statistical analyses.

## 3. Results 

A total of 3096 early-stage NSCLC patients diagnosed at and/or referred to the Department of Oncology at Aarhus University Hospital between 2010 and 2020 were identified. Data on disease stage at diagnosis were available for 2835 patients (91.6%). After excluding patients with stage IA and IIIB, the final study cohort comprised a total of 1341 early-stage (IB-IIIA) NSCLC patients, of whom 55.9% were males, 55% had adenocarcinomas, and most were either current (27.1%) or previous (68.5%) smokers (Table 1). The median age of patients was 71 years (65–77 years).

Of the total 1341 patients, 301 (22.4%) were diagnosed with stage IB, 531 (39.6%) with stage II, and 509 (38.0%) with stage IIIA (Table 1). A total of 563 patients (42.0%) were tested for EGFR mutations (86% of tested patients had adenocarcinomas), with a larger proportion of stage IIIA patients being tested (49.3%) than both stage II (36.7%) and stage IB (38.9%). Among the 563 EGFR-tested patients, 54 (9.6%) were EGFRm^+^ and 509 (90.4%) did not have an EGFR mutation. Among the stage IB patients, 19.7% were EGFRm^+^, while 8.2% and 6.0% were EGFRm^+^ for stages II and IIIA, respectively. Of the total 1341 patients, the PD-L1 expression levels were < 1 for 43.4% of patients, between 1 and 50 for 26.5% of patients, and >50 for 30.1% of patients (866 of patients had missing PD-L1 expression levels since PD-L1 testing was not part of the clinical routine among NSCLC patients until 2015), with no large variations observed across stages or across EGFR mutation status (PD-L1 data stratified by EGFR mutation status not shown). Performance status and histology were similar across all stages (Table 1).

The median follow-up from time of diagnosis was 20 months for all patients, and 32, 24, and 12 months for stage IB, II, and IIIB patients, respectively (Table 1). 

**Figure 1:**
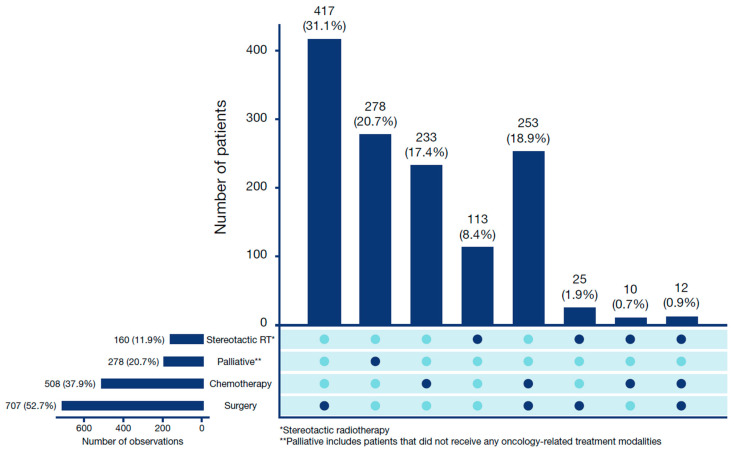
Treatment patterns of all stage IB–IIIA NSCLC patients (N = 1341). Figure legend: The horizontal bars and corresponding numbers for each treatment modality indicate the total number of patients having received this treatment, either alone or in combination with other treatments. The vertical bars and corresponding numbers atop each bar represent the different combinations of treatment modalities. NSCLC = non-small-cell lung cancer.

The most frequent type of treatment was surgery, which was performed in 707 (52.7%) patients, with 417 (31.1%) patients receiving surgery alone and 253 (18.9%) patients receiving a combination of surgery and chemotherapy (Figure 1). Stereotactic body radiation therapy was administered to 160 (11.9%) patients, with 113 (8.4%) patients receiving only SBRT and 10 (0.7%) patients receiving SBRT in combination with chemotherapy (Figure 1; Table 1). Surgery was performed in most patients with stages IB (58.8%) and II (62.5%), whereas 38.9% of stage IIIA patients were treated with surgery either alone or in combination with other treatments. Stereotactic body radiation therapy was predominantly administered to patients with stages IB (25.9%) and II (12.4%) and only to 3.1% of stage IIIA patients (Table 1). Metastases in the CNS occurred in 2.0% of stage IB, 4.1% of stage II, and 6.3% of stage IIIA patients (Table 1) during follow-up.

**Figure 2:**
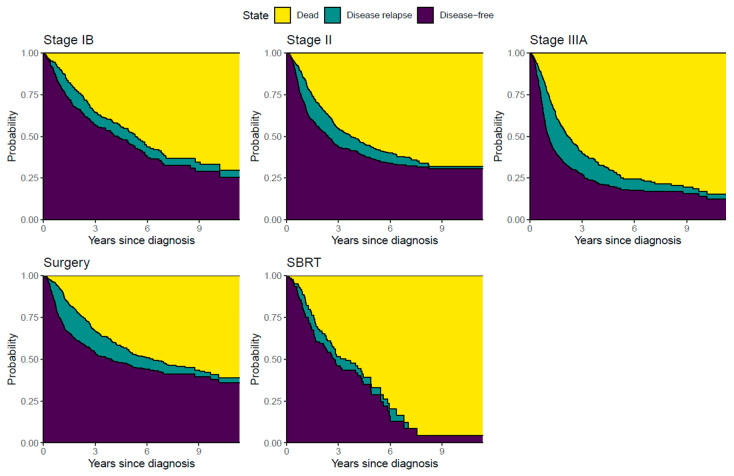
Cumulative probability (risk) of clinical outcomes. Figure legend: Risk of clinical outcomes includes disease-free, disease relapse, or death and is shown by NSCLC stage IB (n = 301), II (n = 531), and IIIA (n = 509) and for patients receiving curative-intended therapy by surgery alone or in combination with chemotherapy (n = 670) and by SBRT alone or in combination with chemotherapy (n = 123). This is a multi-state model where individuals can transfer between the clinical outcomes (e.g., from being disease-free to experiencing disease relapse and to dying), and therefore the same individual can appear in several of the colors/clinical outcomes. NSCLC = non-small-cell lung cancer; SBRT = stereotactic body radiation therapy.

**Figure 3:**
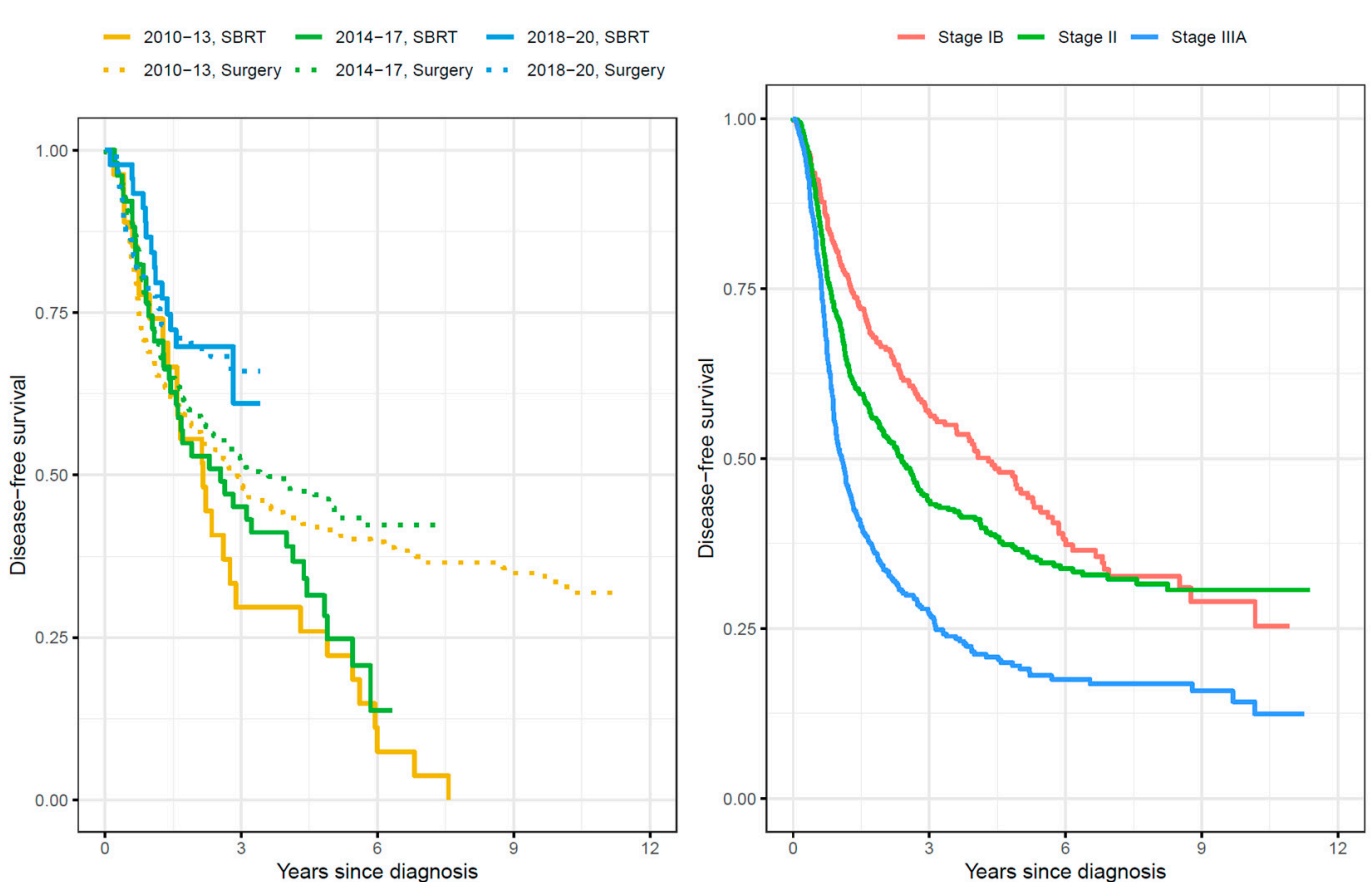
Cumulative probability (risk) of disease-free survival. Figure legend: Disease-free survival is shown by NSCLC stage IB (n = 301), II (n = 531) and IIIA (n = 509) and for patients receiving curative-intended therapy by year of diagnosis and by surgery alone or in combination with chemotherapy (n = 670) and by SBRT alone or in combination with chemotherapy (n = 123). NSCLC = non-small-cell lung cancer; SBRT = stereotactic body radiation therapy.

Patients diagnosed with stages IB and II had more favorable clinical outcomes (i.e., higher probabilities of being disease-free and lower probabilities of disease relapse and death) than stage IIIA patients (Figure 2). Overall survival probabilities 5 years post-NSCLC diagnosis were 70% (95% CI: 63–78) for patients in stage IB; 68% (95% CI: 62–74) for patients in stage II; and 63% (95% CI: 53–73) for stage IIIA patients. The probabilities of DFS 5 years post-NSCLC diagnosis were 49% (95% CI: 43–57) for patients in stage IB; 42% (95% CI: 37–47) for patients in stage II; and 22% (95% CI: 18–28%) for stage IIIA patients (Figure 2 and Figure 3). Similar DFS probabilities were observed for stage IB and II patients from approximately 7 years post-NSCLC diagnosis, whereas DFS among stage IIIA patients remained considerably lower during the entire follow-up period (Figure 3).

Patients undergoing surgery (alone or in combination with chemotherapy) had more favorable clinical outcomes during follow-up (i.e., higher probabilities of being disease-free and lower probabilities of disease relapse and death) than patients treated with SBRT (alone or in combination with chemotherapy) (Figure 2 and Figure 3). Importantly, the probability of DFS increased substantially from 2010 to 2020 (Figure 3). This trend was also observed both in patients treated with surgery (Supplementary Table S1) or with SBRT (Supplementary Table S2). Among patients receiving surgery, the cumulative probability of DFS was 44% higher for patients diagnosed during 2018–2020 than those diagnosed during 2010–2013 (Hazard Ratio (HR): 0.56; 95% CI: 0.40–0.79; Supplementary Table S1). The probability of DFS among patients treated with SBRT was 53% higher for patients diagnosed during 2018–2020 than those diagnosed during 2010–2013 (HR: 0.47; 95% CI: 0.23–0.95; Supplementary Table S2). Disease-free survival did not vary significantly by sex, EGFR mutation status, or histology (Supplementary Tables S1 and S2). For example, the 5-year DFS was 44% (95% CI: 31–63%) for all EGFRm+ patients, 36% (95%: 0.30–0.42%) for EGFRwt, and 38% (95% 0.34–0.42%) for patients not tested for EGFR, which is also illustrated in Supplementary Figure S1.

**Figure 4:**
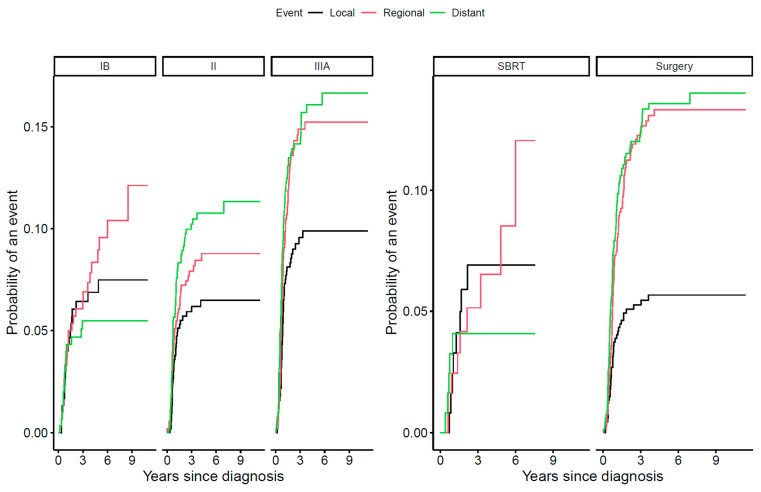
Cumulative incidence of disease relapse and location. Figure legend: Disease relapse is shown by NSCLC stage IB (n = 301), II (n = 531), and IIIA (n = 509) and for patients receiving curative-intended therapy by year of diagnosis and by surgery alone or in combination with chemotherapy (n = 670) and by SBRT alone or in combination with chemotherapy (n = 123). NSCLC = non-small-cell lung cancer; SBRT = stereotactic body radiation therapy.

The cumulative probability of disease relapse during follow-up was higher among stage IIIA than stage IB and II patients. At 3 years from diagnosis, approximately 15% of stage IIIA patients experienced a regional relapse and 15% a distant relapse (Figure 4). Moreover, despite receiving curative-intended surgery at the time of diagnosis, operated stage IB–IIIA patients had relatively high probabilities of regional and distant relapses (Figure 4). In contrast, stage IB–IIIA patients treated with SBRT had a low probability of distant relapse (Figure 4).

## 4. Discussion

This is, to our knowledge, the first study to focus on DFS dynamics and treatment in a large unselected early-stage NSCLC patient population [9,20,21]. In this cohort study of 1341 early-stage NSCLC patients in Denmark, we found that over one-third (42%) of patients were EGFR-tested, and 10% of these were EGFRm^+^. Half (50%) of all patients underwent surgery, either surgery alone or in combination with chemotherapy, whilst SBRT was less common, only being administered to 9% of patients, either alone or in combination with chemotherapy. The probabilities of DFS 5 years post-diagnosis ranged from 49% to 22% across stage IB, II, and IIIA patients. Notably, DFS increased substantially during the entire study period, and similar trends were observed for patients treated with surgery and SBRT. Regional and distant relapses occurred more often among patients with stage IIIA NSCLC than patients with earlier stages of disease. At 3 years post-diagnosis, approximately 40% of stage IIIA patients had experienced a relapse (~10% local, ~15% regional, and ~15% distant relapse).

Our reported OS estimates are in line with previous studies of early-stage NSCLC [4,22], including data from 20 countries with 5-year survival ranging between 60 and 90% among stage I NSCLC patients and 12–40% among stage III NSCLC patients [7]. Our observation of substantially improved clinical outcomes over time among early-stage NSCLC patients during the study period (2010–2020) has also been reported in previous studies [2,23], including the Danish SCAN-LEAF study, which showed that 1- and 2-year OS improved during the study period from 2005 to 2015 [23]. This likely highlights the evolution and success of earlier detection through incidental pulmonary nodule follow-up, as well as better surgical techniques and therapeutic advances with targeted drugs for the management of NSCLC. However, in the annual reports from the Danish Lung Cancer Registry covering Aarhus University Hospital (similar catchment area as in our study), we were not able to observe changes in the diagnostic workup between 2010 and 2018 in relation to the proportion of patients that had a PET scan (94% to 97%), pTNM/cTNM stage shift for surgical patients (7.2% to 6.2%), and median time to diagnosis (27 days to 24 days) [24].

Our study population of early-stage NSCLC patients is a highly heterogeneous group with diverse tumor characteristics and multiple possible treatment options. The EGFR mutation status plays an important role in NSCLC oncogenesis. A population-based cohort study of stage I–IIIA NSCLC patients diagnosed in Denmark during 2013 and 2018 reported that nearly half of the patients (46.5%) underwent EGFR testing with 8.9% identified as EGFRm^+^ [4]. This is comparable to our study, where 42.0% were EGFR-tested and 9.6% were identified as EGFRm^+^. Moreover, our findings reflect the EGFR testing prevalence reported in routine clinical practice in Europe [25]. In general, the uptake of routine EGFR testing with next-generation sequencing has increased in recent years [26] so it is expected that the uptake of routine NGS will result in a slightly higher frequency of detected EGFRm+ patients compared to this cohort diagnosed during 2010–2020.

Regarding the treatment patterns in our cohort, we found that surgical tumor resection was the predominant choice of treatment, consistent with what has previously been reported [23]. For early-stage NSCLC patients who are deemed medically inoperable or those who decline surgery, SBRT has been widely and increasingly implemented since 2012 as the treatment option to achieve tumor control [27]. Recently, increased utilization of SBRT for curative-intended therapy has been associated with improved survival in early-stage NSCLC patients [28]. Moreover, a Danish study evaluating the effect of SBRT on OS for patients with localized NSCLC found a 5-year OS probability of 44% and higher survival among patients treated with SBRT than those who received no treatment [29]. Furthermore, a recent systematic literature review and meta-analysis reported that surgical resection was superior to SBRT with regard to clinical outcomes (i.e., OS and DFS) [30]. Similarly, we found that SBRT was predominantly administered to patients in stage IB (25.9%). However, patients treated with SBRT are typically frailer and older and have more comorbidities and poor performance status, which leaves them ineligible for surgery. Thus, our findings comparing patients treated with surgery vs. SBRT should be interpreted with caution considering patient heterogeneity and potential selection bias.

The development of CNS metastases is a well-known and detrimental complication of NSCLC [31]. In our study population, 4.5% of all patients developed CNS metastases with increasing prevalence by higher disease stage, which reflects previous studies [32]. However, brain imaging was only performed when patients displayed symptoms of brain metastasis; it was not part of the diagnostic workup. Consequently, the number of CNS metastases could be higher due to subclinical lesions. The potential benefit of including brain imaging in the diagnostic workup is highlighted by the emergence of several new treatment modalities that impact the treatment of CNS metastases. These include both SBRT and systemic treatments. For example, the ADAURA study reported an 82% reduction in risk of CNS-related disease relapse or death among stage IB–IIIA NSCLC patients who were treated with osimertinib post-surgery (with or without adjuvant chemotherapy) vs. the placebo (HR: 0.18; 95% CI: 0.10–0.33) [16]. The emergence of immunotherapy for early-stage perioperative NSCLC patients further highlights the need for additional CNS evidence that supports the role of novel treatment paradigms in eradicating micro-metastasis or preventing spread from primary lung lesions in the early-stage setting.

We observed similar relapse dynamics across disease stages, including a considerable proportion of resected patients experiencing regional and distant relapses. Moreover, approximately 40% of stage IIIA patients experienced a relapse 3 years post-diagnosis that varied in location, with local relapse being less common (10%) than both regional (15%) and distant relapses (15%). This agrees with previous reports that underscore the monumental challenge of disease relapse in these patients [11,33]. Fewer patients treated with osimertinib in the ADAURA study had local/regional and distant relapses than placebo-treated patients [17], which highlights the advance that this novel therapy may offer NSCLC patients. Earlier studies estimated that 10–50% of patients treated for early-stage resectable NSCLC will develop locoregional relapse [34]. Considering the poor survival following disease relapse, there is a clear need for improved treatment modalities and strategies to prevent relapse [34], as our study also suggests. Therefore, correct and elaborative diagnostic workup in the early setting will be critical in guiding decision making to uncover driver mutations and interrogating the tumor microenvironment. Encouragingly, we see an increasing number of effective personalized treatments towards oncogenic drivers and novel combination treatments in the perioperative NSCLC setting [35,36,37], as well as an increasing demand to select patient populations based on the molecular footprint of residual disease post-surgery and neo/adjuvant treatments in this maturing era of personal medicine. 

The main strength of our study is the long-term follow-up of an unselected cohort of stage IB-IIIA NSCLC patients. Furthermore, our use of comprehensive and high-quality clinical data (including treatment information from electronic medical records) with mandatory registration by the treating physician as well as reliable registry data on vital status and accurate linkage across data sources together improve the external validity of our findings and limit the risk of selection bias and loss to follow-up.

These findings should, however, be interpreted with some caution, particularly the stratified analyses based on few observations. For example, the finding that SBRT-treated stage IB–IIIA patients had a low probability of distant relapse was based on a small number of patients, which may result in less precise effect estimates. Furthermore, this single-center study only included patients diagnosed at and/or referred for cancer treatment to the Department of Oncology at Aarhus University Hospital, although we have no reason to believe that our results do not represent a nationwide perspective. Lastly, in the analyses stratified by treatment modality (surgery vs. SBRT), we excluded palliative patients who were defined as patients who received no initial oncology-related treatment modalities (i.e., surgery, chemotherapy, or SBRT), which might also have included patients with missing treatment information.

## 5. Conclusions

This real-world evidence study reveals the current clinical management and disease recurrence patterns of early-stage NSCLC patients in Denmark. These data enable clinical trials on new perioperative treatment modalities in early-stage NSCLC to be put into perspective and potentially create valuable insights for clinicians to further optimize treatment options and improve clinical outcomes in these patients. Importantly, we found that DFS improved during the study period (2010–2020). The improved DFS over time was observed both for patients selected for surgery and/or SBRT. Nevertheless, over a third of patients treated with surgery experienced disease relapse, which highlights that effective treatments are still needed for early-stage NSCLC. Moreover, the evolving treatment landscape for perioperative NSCLC using personalized oncogenic driver mutations, as well as combination immunotherapy, warrants the thorough upfront diagnosis of patients and optimized patient selection for treatment strategies.

## Figures and Tables

**Table 1:** Descriptive characteristics of all patients (N = 1341) by disease stage.

	Total	Stage IB	Stage II	Stage IIIA	*p*-Value
At baseline					
n (%)	1341	301 (22.4)	531 (39.6)	509 (38.0)	
Median age (IQR)	71 (65–77)	72 (66–78)	71 (65–76)	70 (64–76)	0.014 *
Sex					
Male	749 (55.9)	164 (54.5)	310 (58.4)	275 (54.0)	0.318
Female	592 (44.1)	137 (45.5)	221 (41.6)	234 (46.0)	
Year of diagnosis					
2010–2013	422 (31.5)	106 (35.2)	157 (29.6)	159 (31.2)	0.412
2014–2017	566 (42.2)	126 (41.9)	230 (43.3)	210 (41.3)	
2018–2020	353 (26.3)	69 (22.9)	144 (27.1)	140 (27.5)	
Histology					
Adenocarcinoma	738 (55.0)	181 (60.1)	271 (51.0)	286 (56.2)	0.031
Squamous	523 (39.0)	110 (36.5)	219 (41.2)	194 (38.1)	
Other	80 (6.0)	10 (3.3)	41 (7.7)	29 (5.7)	
Smoking					
Previous smoker	918 (68.5)	206 (68.4)	363 (68.4)	349 (68.6)	0.805
Current smoker	364 (27.1)	78 (25.9)	146 (27.5)	140 (27.5)	
Never smoker	59 (4.4)	17 (5.6)	22 (4.1)	20 (3.9)	
EGFR testing					
No	778 (58.0)	184 (61.1)	336 (63.3)	258 (50.7)	<0.001
Yes	563 (42.0)	117 (38.9)	195 (36.7)	251 (49.3)	
EGFR result among patients tested					
EGFRm^+^	54 (9.6)	23 (19.7)	16 (8.2)	15 (6.0)	<0.001
WT	509 (90.4)	94 (80.3)	179 (91.8)	236 (94.0)	
PD-L1 expression level					
<1	206 (43.4)	38 (45.2)	70 (40.7)	98 (44.7)	0.453
1–50	126 (26.5)	25 (29.8)	51 (29.7)	50 (22.8)	
>50	143 (30.1)	21 (25.0)	51 (29.7)	71 (32.4)	
Unknown (not included in reported %)	866	217	359	290	
Performance Status					
0	237 (27.4)	50 (26.5)	97 (28.2)	90 (27.1)	0.653
1	400 (46.2)	91 (48.1)	146 (42.4)	163 (49.1)	
2+	228 (26.4)	48 (25.4)	101 (29.4)	79 (23.8)	
Unknown (not included in reported %)	476	112	187	177	
During follow-up					
Median follow-up months (IQR)	20 (9–43)	32 (15–59)	24 (10–48)	12 (8–27)	
Surgery **					
Yes	707 (52.7)	177 (58.8)	332 (62.5)	198 (38.9)	
Stereotactic body radiation therapy **^,^ ***					
Yes	160 (11.9)	78 (25.9)	66 (12.4)	16 (3.1)	
Chemotherapy **					
Yes	508 (37.9)	27 (9.0)	182 (34.3)	299 (58.7)	
Palliative treatment ****					
Yes	278 (20.7)	43 (14.3)	105 (19.8)	130 (25.5)	
CNS metastases					
Yes	60 (4.5)	6 (2.0)	22 (4.1)	32 (6.3)	
Disease relapse					
No	856 (68.3)	225 (77.9)	370 (73.6)	261 (56.6)	
Yes	397 (31.7)	64 (22.1)	133 (26.4)	200 (43.4)	
Unknown (not included in reported %)	88	12	28	48	
Location of disease relapse					
Local	102 (25.7)	21 (32.8)	33 (24.8)	48 (24.0)	
Regional	145 (36.5)	27 (42.2)	44 (33.1)	74 (37.0)	
Distant	150 (37.8)	16 (25.0)	56 (42.1)	78 (39.0)	
Vital status *****					
Alive	555 (41.4)	151 (50.2)	240 (45.2)	164 (32.2)	
Dead	786 (58.6)	150 (49.8)	291 (54.8)	345 (67.8)	

* Based on Kruskal–Wallis non-parametric test; ** alone or in combination with other treatments (see Figure 1); *** stereotactic body radiation therapy was administered to patients who were either ineligible for surgery or had progressed with oligometastases post-surgery; **** Patients who did not receive any oncology-related treatment modalities (i.e., surgery, chemotherapy, or stereotactic radiotherapy) during follow-up; where EGFR = epidermal growth factor receptor; EGFRm^+^ = epidermal growth factor receptor-positive mutations (EGFRm^+^); IQR = interquartile range; PD-L1 = programmed death-ligand 1; ***** numbers on vital status should be interpreted with caution due to lack of considering time at risk for this descriptive reporting. The overall survival estimates that are reported elsewhere take time at risk into consideration. WT = wild type/no EGFR mutation.

## Data Availability

Data underlying the findings described in this manuscript may be obtained in accordance with AstraZeneca’s data sharing policy described at https://astrazenecagrouptrials.pharmacm.com/ST/Submission/Disclosure (accessed on 23 October 2023).

## Supplementary Materials

The following supporting information can be downloaded at https://www.mdpi.com/article/10.3390/cancers15215130/s1. Table S1. Disease-free survival among Stage IB–IIIA NSCLC patients receiving curative-intended surgery alone or in combination with chemotherapy (N = 670), by risk factors; Table S2. Disease-free survival among Stage IB–IIIA NSCLC patients receiving curative-intended stereotactic body radiation therapy alone or in combination with chemotherapy (N = 123) by risk factors; Figure S1. Cumulative probability (risk) of disease-free survival by EGFR status.
