# Supplementary material for: Diagnostic Workup, Treatment Patterns, and Clinical Outcomes in Early-Stage IB–IIIA Non-Small-Cell Lung Cancer Patients in Denmark
Source: Cancers (Basel). 2023 Oct 25;15(21):5130. doi: 10.3390/cancers15215130 (PMC10647574; doi:10.3390/cancers15215130)
Supplement: Supplementary file 1 [file cancers-15-05130-s001.zip › cancers-2620698-supplementary.pdf]

Supplementary material

Supplementary Table S1. Disease-free survival among Stage IB–IIIA NSCLC patients receiving curative-intended surgery alone or in combination with chemotherapy (N=670), by risk factors

Variable		N	Hazard ratio		p
**Age (in years)**		670		1.00 (0.99, 1.02)	0.45
**Sex**	Male	392		Reference	
	Female	278		0.95 (0.76, 1.19)	0.64
**Year**	2010−13	219		Reference	
	2014−17	291		0.83 (0.66, 1.06)	0.13
	2018−20	160		0.56 (0.40, 0.79)	<0.01
**Stage**	IB	169		Reference	
	II	312		1.25 (0.94, 1.66)	0.13
	IIIA	189		2.42 (1.80, 3.24)	<0.01
**EGFR status**	WT	251		Reference	
	EGFRm+	39		0.95 (0.58, 1.54)	0.82
	Not tested	380		0.84 (0.64, 1.10)	0.21
**Histology**	Adenocarcinoma	387		Reference	
	Squamous	239		1.02 (0.77, 1.33)	0.91
	Other	44		0.90 (0.57, 1.42)	0.65

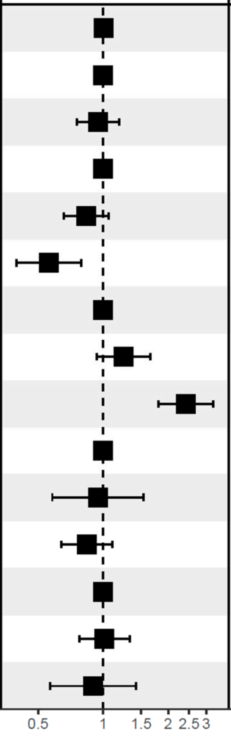

Figure legend:

All risk factors listed in this table are included in the Cox proportional hazard model, i.e., estimates are mutually adjusted. Hazard ratios <1 and point estimates on the left side of the forest plot indicate higher DFS.

Where EGFR-mutation positive ($EGFRm^+$), NSCLC= non-small cell lung cancer, WT=wild type / no EGFR mutation.

Supplementary Table S2. Disease-free survival among Stage IB–IIIA NSCLC patients receiving curative-intended stereotactic body radiation therapy alone or in combination with chemotherapy (N=123) by risk factors

Variable		N	Hazard ratio		p
**Age (in years)**		123		1.01 (0.99, 1.04)	0.36
**Sex**	Male	67		Reference	
	Female	56		1.17 (0.70, 1.96)	0.56
**Year**	2010−13	27		Reference	
	2014−17	51		0.83 (0.48, 1.42)	0.49
	2018−20	45		0.47 (0.23, 0.95)	0.04
**Stage**	IB	70		Reference	
	II	46		1.29 (0.80, 2.07)	0.30
	IIIA	7		3.62 (1.34, 9.77)	0.01
**EGFR status**	WT	40		Reference	
	EGFRm+	8		0.84 (0.27, 2.63)	0.77
	Not tested	75		0.74 (0.37, 1.45)	0.38
**Histology**	Adenocarcinoma	69		Reference	
	Squamous	46		1.62 (0.82, 3.20)	0.17
	Other	8		0.89 (0.28, 2.86)	0.85

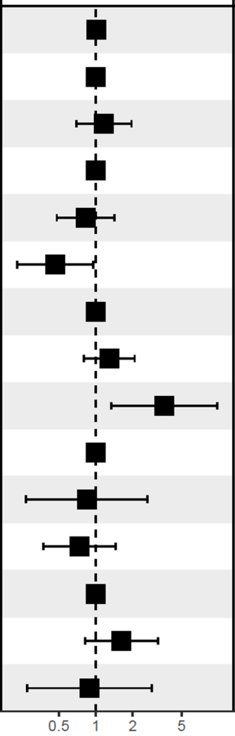

Figure legend:

All risk factors listed in this table are included in the Cox proportional hazard model, i.e., estimates are mutually adjusted. Hazard ratios <1 and point estimates on the left side of the forest plot indicate higher DFS.

Where EGFR-mutation positive (EGFRm+), NSCLC= non-small cell lung cancer, WT=wild type / no EGFR mutation.

Supplementary Figure S1: Cumulative probability (risk) of disease-free survival by EGFR status

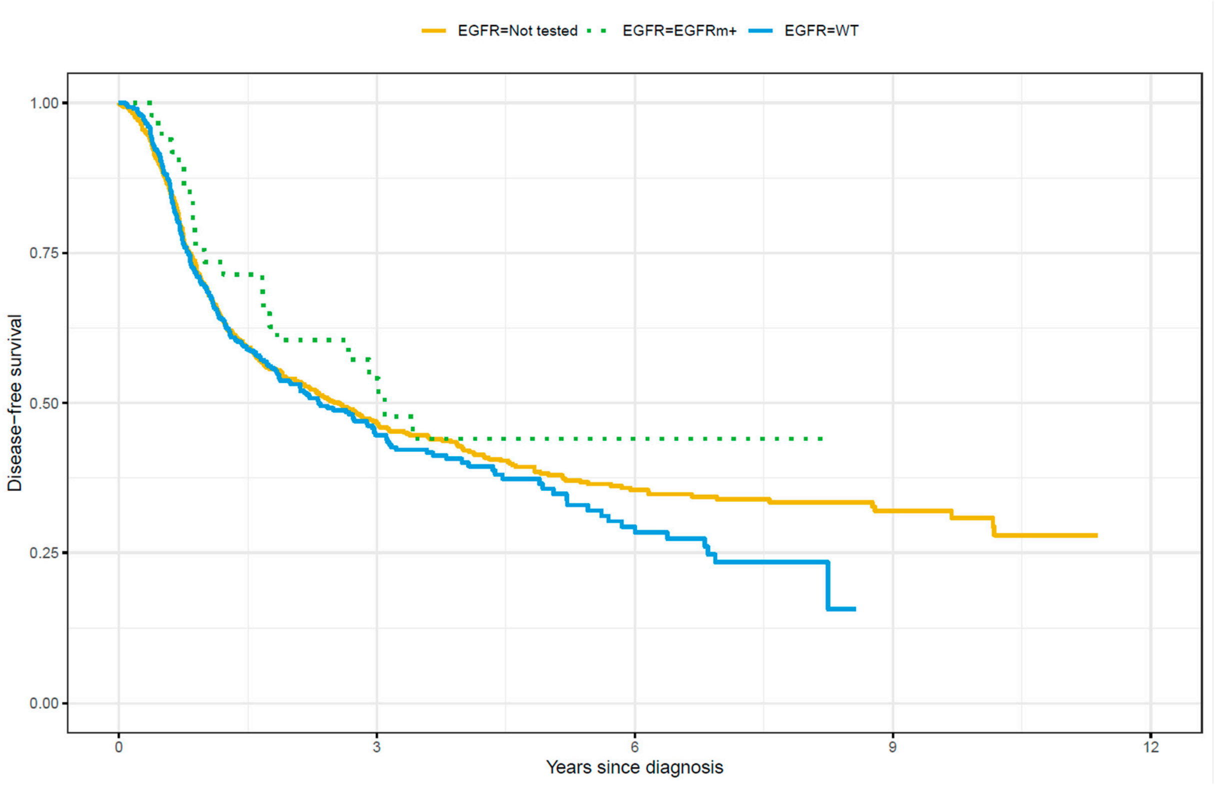

Figure legend:

Disease-free survival is shown by EGFRm+ (n=54), EGFRwt (n=509) and EGFR unknown/patients without EGFR testing (n=778).

Where: EGFR= epidermal growth factor receptor, EGFRm+ = epidermal growth factor receptor-positive mutations, EGFRwt = epidermal growth factor receptor wild type (no EGFR mutation).
